# *N*-Methyl-2-(1,2,3,4-tetra­hydro­naph­thalen-1-yl­idene)hydrazinecarbo­thio­amide

**DOI:** 10.1107/S1600536814003079

**Published:** 2014-02-15

**Authors:** Adriano Bof de Oliveira, Bárbara Regina Santos Feitosa, Christian Näther, Inke Jess

**Affiliations:** a Departamento de Química, Universidade Federal de Sergipe, Av. Marechal Rondon s/n, Campus, 49100-000 São Cristóvão, SE, Brazil; b Institut für Anorganische Chemie, Christian-Albrechts-Universität zu Kiel, Max-Eyth Strasse 2, D-24118 Kiel, Germany

## Abstract

There are two independent mol­ecules in the asymmetric unit of the title compound, C_12_H_15_N_3_S, both of which display disorder of several C atoms in the N-bound ring (occupancy ratios of 0.75:0.25 in the first independent mol­ecule and 0.50:0.50 in the second) with the methyl H atoms also being disordered in the first mol­ecule (occupancy ratio of 0.70:0.30). The planes of the benzene ring and the N—N—C—N fragment make dihedral angles of 12.92 (14)° in the first independent mol­ecule and 7.60 (13)° in the second. In the crystal, mol­ecules are linked by weak N—H⋯S hydrogen bonds into chains along the *a-*axis direction. The crystal packing ressembles a herringbone arrangement.

## Related literature   

For the synthesis, coordination chemistry and biological activity of thio­semicarbazones, see: Lobana *et al.* (2009 ▶). For one of the first reports of the synthesis of thio­semicarbazone derivatives, see: Freund & Schander (1902 ▶).
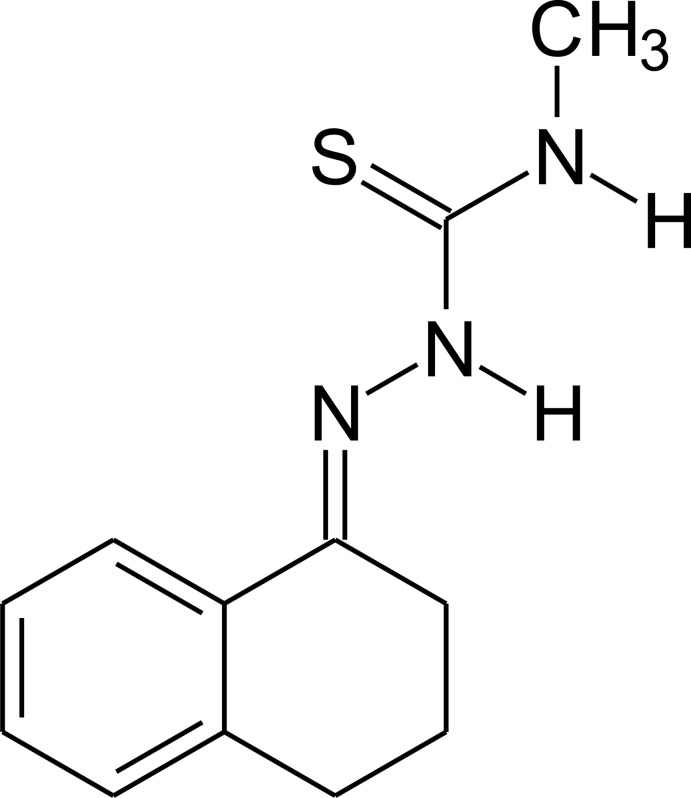


## Experimental   

### 

#### Crystal data  

C_12_H_15_N_3_S
*M* *_r_* = 233.33
Triclinic, 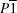

*a* = 10.6234 (6) Å
*b* = 10.9425 (6) Å
*c* = 11.0576 (6) Å
α = 73.685 (4)°
β = 79.152 (4)°
γ = 88.614 (4)°
*V* = 1211.04 (12) Å^3^
*Z* = 4
Mo *K*α radiation
μ = 0.24 mm^−1^
*T* = 200 K
0.2 × 0.1 × 0.1 mm

#### Data collection  

Stoe IPDS-1 diffractometer
11521 measured reflections
5125 independent reflections
4111 reflections with *I* > 2σ(*I*)
*R*_int_ = 0.031

#### Refinement  

*R*[*F* ^2^ > 2σ(*F* ^2^)] = 0.041
*wR*(*F* ^2^) = 0.114
*S* = 1.06
5125 reflections
334 parameters
H atoms treated by a mixture of independent and constrained refinement
Δρ_max_ = 0.42 e Å^−3^
Δρ_min_ = −0.42 e Å^−3^

### 

Data collection: *X-AREA* (Stoe & Cie, 2008 ▶); cell refinement: *X-AREA*; data reduction: *X-RED32* (Stoe & Cie, 2008 ▶); program(s) used to solve structure: *SHELXS97* (Sheldrick, 2008 ▶); program(s) used to refine structure: *SHELXL97* (Sheldrick, 2008 ▶); molecular graphics: *DIAMOND* (Brandenburg, 2006 ▶); software used to prepare material for publication: *publCIF* (Westrip, 2010 ▶).

## Supplementary Material

Crystal structure: contains datablock(s) I. DOI: 10.1107/S1600536814003079/bt6960sup1.cif

Structure factors: contains datablock(s) I. DOI: 10.1107/S1600536814003079/bt6960Isup2.hkl

Supporting information file. DOI: 10.1107/S1600536814003079/bt6960Isup3.cml

CCDC reference: 986205

Additional supporting information:  crystallographic information; 3D view; checkCIF report

## Figures and Tables

**Table 1:** Hydrogen-bond geometry (Å, °)

*D*—H⋯*A*	*D*—H	H⋯*A*	*D*⋯*A*	*D*—H⋯*A*
N3—H3⋯S21^i^	0.88 (2)	3.03 (2)	3.7226 (15)	138.0

Symmetry code: (i) 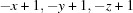.

**Fig. 1.**
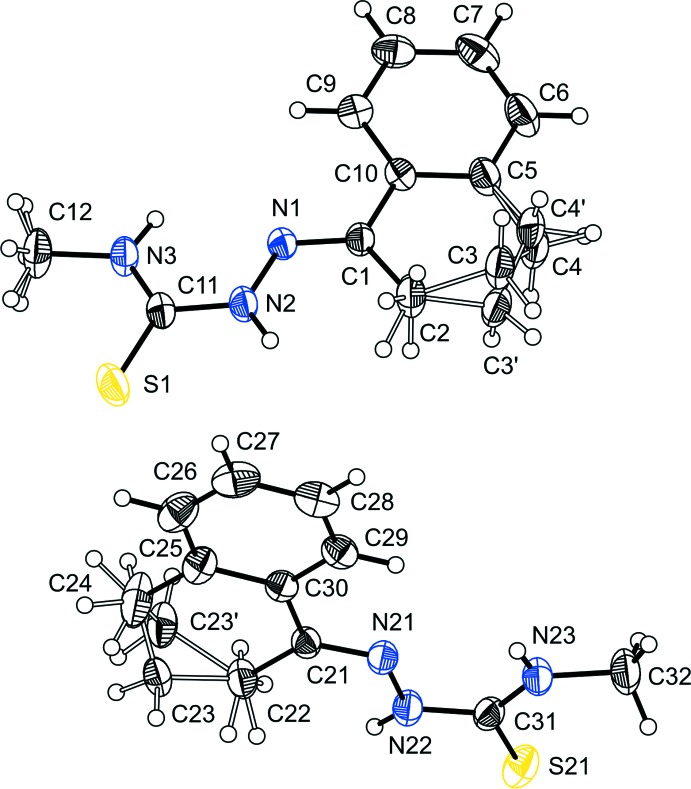
The molecular structure of the title compound with labeling and displacement ellipsoids drawn at the 40% probability level. Disorder is shown with full and open bonds.

**Fig. 2.**
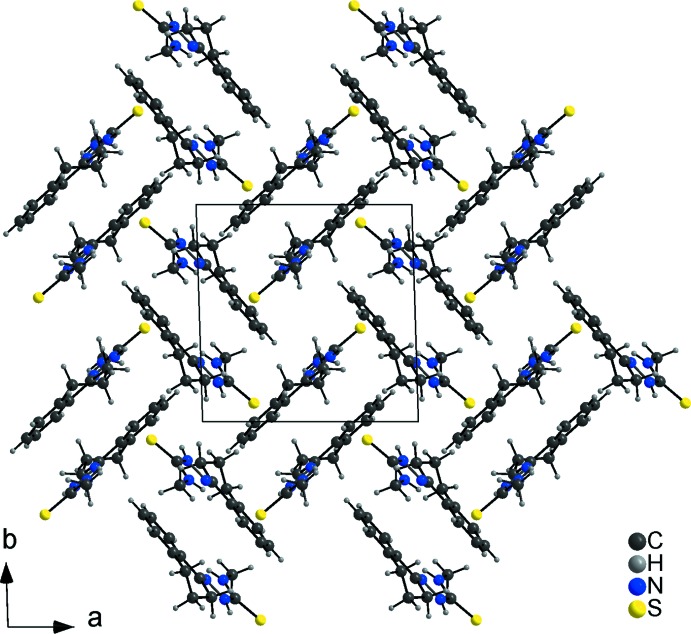
: Crystal structure of the title compound viewed along the c-axis. The disorder is not shown for clarity.

## supplementary crystallographic information

### 1. Comment

Thiosemicarbazone derivatives have been used as ligands in coordination chemistry as well as for applications in biological systems, because some of them shows antifungal, antibacterial and anticancer properties (Lobana *et al.*, 2009). As part of our study on thiosemicarbazone compounds, we report herein the crystal structure of a tetralone thiosemicarbazone derivative.

In the crystal structure of the title compound, there are two crystallographically independent molecules on the asymmetric unit in general positions, in which some of the C, and H atoms are disordered (Fig. 1). For one crystallographic independent molecule, the dihedral angle between the benzene ring (C5—C10) and the N1—N2—C11—N3 fragment amounts to 12.92 (14)° and for the second crystallographically independent molecule the dihedral angle between the benzene ring (C25—C30) and the N21—N22—C31—N23 fragment amouts to 7.60 (13)°. So, the molecules do not only show a different kind of disorder, but differ also in the molecular geometry.

In the crystal, the molecules are linked by long weak N—H···S hydrogen bonding into chains that run along the *a*-axis. The S···H distances amount to 3.02 (2) Å for the S1 and 3.291 (22) Å for the S21 atoms (Table 1). When viewed along the *c*-axis (Fig. 2), the molecules show a herringbone motif.

### 2. Experimental

Starting materials were commercially available and were used without further purification. The tetralone-thiosemicarbazone derivative synthesis was adapted from a procedure reported previously (Freund & Schander, 1902). The hydrochloric acid catalyzed reaction of tetralone (8,83 mmol) and 4-methylthiosemicarbazide (8,83 mmol) in ethanol (50 ml) was refluxed for 6 h. After cooling and filtering, the title compound was obtained as a solid. Crystals suitable for X-ray diffraction of the title compound were obtained in tethahydrofuran by the slow evaporation of solvent.

### 3. Refinement

All non-hydrogen atoms were refined anisotropic. All H atoms were located in difference map but were positioned with idealized geometry (methyl H atoms allowed to rotate but no to tip) and were refined isotropic with *U*_iso_(H) = 1,2 *U*_eq_(C) (1,5 for methyl H atoms) using a riding model with C—H = 0,95 Å for aromatic and C—H = 0,99 Å for methylene. The N—H H atoms were located in difference map and refined with varying coordinates and varying isotropic displacement parameters. There are two crystallographically independent molecules in the asymmetric unit. In both of them several C atoms disordered (C3, C4 and C23) and were refined using a split model. The methyl C12 H atoms are disordered and were refined in two orientations.

### Crystal data

**Table:**

C_12_H_15_N_3_S	*Z* = 4
*M**_r_* = 233.33	*F*(000) = 496
Triclinic, *P*1	*D*_x_ = 1.280 Mg m^−^^3^
Hall symbol: -P 1	Mo *K*α radiation, λ = 0.71073 Å
*a* = 10.6234 (6) Å	Cell parameters from 11521 reflections
*b* = 10.9425 (6) Å	θ = 1.9–27.0°
*c* = 11.0576 (6) Å	µ = 0.24 mm^−^^1^
α = 73.685 (4)°	*T* = 200 K
β = 79.152 (4)°	Block, white
γ = 88.614 (4)°	0.2 × 0.1 × 0.1 mm
*V* = 1211.04 (12) Å^3^	

### Data collection

**Table:**

Stoe IPDS-1 diffractometer	4111 reflections with *I* > 2σ(*I*)
Radiation source: fine-focus sealed tube, Stoe IPDS-1	*R*_int_ = 0.031
Graphite monochromator	θ_max_ = 27.0°, θ_min_ = 1.9°
φ scans	*h* = −13→12
11521 measured reflections	*k* = −13→13
5125 independent reflections	*l* = −14→13

### Refinement

**Table:**

Refinement on *F*^2^	Primary atom site location: structure-invariant direct methods
Least-squares matrix: full	Secondary atom site location: difference Fourier map
*R*[*F*^2^ > 2σ(*F*^2^)] = 0.041	Hydrogen site location: inferred from neighbouring sites
*wR*(*F*^2^) = 0.114	H atoms treated by a mixture of independent and constrained refinement
*S* = 1.06	*w* = 1/[σ^2^(*F*_o_^2^) + (0.0541*P*)^2^ + 0.2784*P*] where *P* = (*F*_o_^2^ + 2*F*_c_^2^)/3
5125 reflections	(Δ/σ)_max_ = 0.001
334 parameters	Δρ_max_ = 0.42 e Å^−^^3^
0 restraints	Δρ_min_ = −0.42 e Å^−^^3^

### Special details

**Table:**

Geometry. All e.s.d.'s (except the e.s.d. in the dihedral angle between two l.s. planes) are estimated using the full covariance matrix. The cell e.s.d.'s are taken into account individually in the estimation of e.s.d.'s in distances, angles and torsion angles; correlations between e.s.d.'s in cell parameters are only used when they are defined by crystal symmetry. An approximate (isotropic) treatment of cell e.s.d.'s is used for estimating e.s.d.'s involving l.s. planes.
Refinement. Refinement of *F*^2^ against ALL reflections. The weighted *R*-factor *wR* and goodness of fit *S* are based on *F*^2^, conventional *R*-factors *R* are based on *F*, with *F* set to zero for negative *F*^2^. The threshold expression of *F*^2^ > σ(*F*^2^) is used only for calculating *R*-factors(gt) *etc*. and is not relevant to the choice of reflections for refinement. *R*-factors based on *F*^2^ are statistically about twice as large as those based on *F*, and *R*- factors based on ALL data will be even larger.

### Fractional atomic coordinates and isotropic or equivalent isotropic displacement parameters (Å^2^)

**Table:**

	*x*	*y*	*z*	*U*_iso_*/*U*_eq_	Occ. (<1)
C1	0.48672 (15)	0.18650 (14)	0.58968 (14)	0.0359 (3)	
C2	0.5663 (2)	0.2436 (2)	0.65971 (16)	0.0585 (5)	
H2A	0.5465	0.3348	0.6461	0.070*	0.75
H2B	0.6581	0.2385	0.6229	0.070*	0.75
H2C	0.5993	0.3292	0.6057	0.070*	0.25
H2D	0.6406	0.1896	0.6772	0.070*	0.25
C3	0.5434 (3)	0.1769 (3)	0.8050 (2)	0.0523 (6)	0.75
H3A	0.5785	0.0904	0.8205	0.063*	0.75
H3B	0.5878	0.2259	0.8483	0.063*	0.75
C4	0.4004 (7)	0.1680 (7)	0.8593 (8)	0.0515 (13)	0.75
H4A	0.3634	0.2536	0.8400	0.062*	0.75
H4B	0.3844	0.1303	0.9535	0.062*	0.75
C3'	0.4840 (13)	0.2542 (11)	0.7888 (8)	0.074 (3)	0.25
H3C	0.5398	0.2787	0.8411	0.088*	0.25
H3D	0.4196	0.3207	0.7715	0.088*	0.25
C4'	0.420 (3)	0.133 (2)	0.858 (3)	0.071 (7)	0.25
H4C	0.4868	0.0699	0.8806	0.085*	0.25
H4D	0.3683	0.1411	0.9392	0.085*	0.25
C5	0.34020 (17)	0.08243 (17)	0.79474 (15)	0.0456 (4)	
C6	0.24021 (19)	−0.00422 (19)	0.86362 (18)	0.0570 (5)	
H6	0.2117	−0.0132	0.9525	0.068*	
C7	0.18239 (19)	−0.07657 (18)	0.8054 (2)	0.0582 (5)	
H7	0.1152	−0.1359	0.8542	0.070*	
C8	0.22184 (17)	−0.06331 (16)	0.67515 (19)	0.0497 (4)	
H8	0.1809	−0.1126	0.6345	0.060*	
C9	0.32074 (15)	0.02177 (14)	0.60490 (16)	0.0396 (3)	
H9	0.3479	0.0307	0.5159	0.048*	
C10	0.38118 (15)	0.09484 (13)	0.66391 (14)	0.0350 (3)	
N1	0.50410 (13)	0.21270 (11)	0.46681 (12)	0.0354 (3)	
N2	0.60085 (15)	0.29850 (14)	0.39563 (12)	0.0430 (3)	
H2	0.652 (2)	0.3371 (19)	0.431 (2)	0.056 (6)*	
C11	0.62069 (17)	0.32887 (15)	0.26515 (14)	0.0420 (4)	
S1	0.73841 (6)	0.43493 (5)	0.17704 (4)	0.06437 (17)	
N3	0.54320 (15)	0.27078 (15)	0.21718 (13)	0.0441 (3)	
H3	0.486 (2)	0.215 (2)	0.272 (2)	0.057 (6)*	
C12	0.5478 (2)	0.2904 (2)	0.08134 (17)	0.0624 (5)	
H12A	0.4843	0.2334	0.0693	0.094*	0.70
H12B	0.6336	0.2719	0.0410	0.094*	0.70
H12C	0.5285	0.3790	0.0418	0.094*	0.70
H12D	0.6133	0.3561	0.0321	0.094*	0.30
H12E	0.4640	0.3177	0.0604	0.094*	0.30
H12F	0.5691	0.2105	0.0596	0.094*	0.30
C21	1.03532 (14)	0.72157 (13)	0.43323 (14)	0.0345 (3)	
C22	0.98459 (18)	0.80910 (17)	0.32295 (15)	0.0463 (4)	
H22A	0.9799	0.8963	0.3330	0.056*	0.50
H22B	0.8968	0.7801	0.3238	0.056*	0.50
H22C	1.0370	0.8893	0.2905	0.056*	0.50
H22D	0.8954	0.8305	0.3536	0.056*	0.50
C23	1.0710 (4)	0.8118 (3)	0.1933 (3)	0.0439 (7)	0.50
H23A	1.0273	0.8583	0.1231	0.053*	0.50
H23B	1.1517	0.8596	0.1847	0.053*	0.50
C23'	0.9870 (4)	0.7468 (4)	0.2094 (3)	0.0527 (9)	0.50
H23C	0.9161	0.6824	0.2339	0.063*	0.50
H23D	0.9720	0.8135	0.1324	0.063*	0.50
C24	1.1012 (3)	0.6896 (3)	0.1790 (2)	0.0752 (7)	
H24A	1.1638	0.6994	0.0981	0.090*	0.50
H24B	1.0225	0.6481	0.1713	0.090*	0.50
H24C	1.1662	0.7578	0.1307	0.090*	0.50
H24D	1.0891	0.6392	0.1202	0.090*	0.50
C25	1.15584 (17)	0.60384 (17)	0.28740 (17)	0.0451 (4)	
C26	1.23846 (19)	0.50776 (19)	0.2683 (2)	0.0562 (5)	
H26	1.2618	0.4967	0.1848	0.067*	
C27	1.28696 (19)	0.42841 (18)	0.3681 (2)	0.0571 (5)	
H27	1.3434	0.3633	0.3533	0.069*	
C28	1.25370 (17)	0.44333 (17)	0.4896 (2)	0.0518 (4)	
H28	1.2872	0.3885	0.5585	0.062*	
C29	1.17199 (15)	0.53750 (15)	0.51113 (17)	0.0424 (4)	
H29	1.1488	0.5468	0.5953	0.051*	
C30	1.12244 (14)	0.61990 (14)	0.41059 (14)	0.0352 (3)	
N21	1.00717 (13)	0.72885 (12)	0.54928 (12)	0.0378 (3)	
N22	0.92325 (15)	0.81998 (13)	0.57445 (13)	0.0417 (3)	
H22	0.883 (2)	0.8683 (19)	0.517 (2)	0.055 (6)*	
C31	0.87550 (17)	0.81552 (16)	0.69932 (15)	0.0420 (4)	
S21	0.76008 (5)	0.91523 (5)	0.73333 (5)	0.05696 (15)	
N23	0.92760 (17)	0.73086 (15)	0.78559 (13)	0.0475 (3)	
H23	0.985 (2)	0.683 (2)	0.762 (2)	0.059 (6)*	
C32	0.8857 (2)	0.7063 (2)	0.92353 (17)	0.0637 (6)	
H32A	0.8908	0.7858	0.9470	0.096*	
H32B	0.9411	0.6437	0.9682	0.096*	
H32C	0.7969	0.6730	0.9483	0.096*	

### Atomic displacement parameters (Å^2^)

**Table:**

	*U*^11^	*U*^22^	*U*^33^	*U*^12^	*U*^13^	*U*^23^
C1	0.0370 (8)	0.0407 (7)	0.0292 (7)	−0.0037 (6)	−0.0035 (6)	−0.0102 (6)
C2	0.0602 (12)	0.0828 (13)	0.0308 (8)	−0.0339 (10)	−0.0013 (8)	−0.0151 (8)
C3	0.0488 (15)	0.0784 (19)	0.0313 (11)	−0.0160 (13)	−0.0046 (10)	−0.0188 (12)
C4	0.056 (2)	0.069 (4)	0.0309 (16)	−0.015 (2)	0.0027 (16)	−0.022 (2)
C3'	0.106 (9)	0.081 (6)	0.036 (4)	−0.049 (6)	0.009 (5)	−0.029 (4)
C4'	0.107 (14)	0.068 (12)	0.031 (5)	−0.022 (8)	0.022 (7)	−0.023 (7)
C5	0.0455 (10)	0.0531 (9)	0.0329 (8)	−0.0093 (7)	−0.0038 (7)	−0.0048 (7)
C6	0.0531 (11)	0.0669 (12)	0.0377 (9)	−0.0157 (9)	−0.0003 (8)	0.0026 (8)
C7	0.0479 (11)	0.0510 (10)	0.0615 (12)	−0.0149 (8)	−0.0065 (9)	0.0059 (8)
C8	0.0417 (9)	0.0410 (8)	0.0666 (11)	−0.0041 (7)	−0.0151 (9)	−0.0117 (8)
C9	0.0355 (8)	0.0406 (8)	0.0439 (8)	0.0026 (6)	−0.0093 (7)	−0.0127 (6)
C10	0.0343 (8)	0.0354 (7)	0.0327 (7)	0.0005 (6)	−0.0065 (6)	−0.0052 (6)
N1	0.0377 (7)	0.0373 (6)	0.0296 (6)	−0.0036 (5)	−0.0033 (5)	−0.0084 (5)
N2	0.0479 (8)	0.0508 (8)	0.0276 (6)	−0.0152 (6)	−0.0011 (6)	−0.0093 (5)
C11	0.0476 (10)	0.0462 (8)	0.0291 (7)	−0.0023 (7)	−0.0021 (7)	−0.0088 (6)
S1	0.0763 (4)	0.0707 (3)	0.0348 (2)	−0.0303 (3)	0.0012 (2)	−0.0016 (2)
N3	0.0461 (8)	0.0577 (8)	0.0271 (6)	−0.0038 (7)	−0.0032 (6)	−0.0119 (6)
C12	0.0653 (13)	0.0932 (15)	0.0298 (8)	−0.0070 (11)	−0.0084 (8)	−0.0189 (9)
C21	0.0342 (8)	0.0374 (7)	0.0304 (7)	−0.0045 (6)	−0.0047 (6)	−0.0078 (6)
C22	0.0542 (11)	0.0497 (9)	0.0317 (8)	0.0123 (8)	−0.0068 (7)	−0.0079 (7)
C23	0.0446 (19)	0.0546 (19)	0.0265 (14)	−0.0051 (15)	−0.0023 (13)	−0.0039 (13)
C23'	0.059 (2)	0.069 (2)	0.0328 (16)	0.016 (2)	−0.0152 (16)	−0.0156 (16)
C24	0.0950 (18)	0.0995 (17)	0.0404 (10)	0.0374 (14)	−0.0212 (11)	−0.0322 (11)
C25	0.0414 (9)	0.0532 (9)	0.0446 (9)	0.0016 (7)	−0.0067 (7)	−0.0210 (7)
C26	0.0478 (11)	0.0641 (11)	0.0641 (12)	0.0030 (9)	−0.0033 (9)	−0.0347 (10)
C27	0.0423 (10)	0.0481 (10)	0.0848 (14)	0.0060 (8)	−0.0100 (10)	−0.0267 (9)
C28	0.0390 (9)	0.0434 (9)	0.0697 (12)	0.0016 (7)	−0.0139 (9)	−0.0085 (8)
C29	0.0361 (9)	0.0424 (8)	0.0460 (9)	−0.0040 (6)	−0.0085 (7)	−0.0073 (7)
C30	0.0284 (7)	0.0380 (7)	0.0385 (8)	−0.0057 (6)	−0.0039 (6)	−0.0107 (6)
N21	0.0401 (7)	0.0409 (7)	0.0331 (6)	0.0004 (5)	−0.0065 (5)	−0.0118 (5)
N22	0.0489 (8)	0.0447 (7)	0.0316 (6)	0.0046 (6)	−0.0058 (6)	−0.0123 (5)
C31	0.0448 (9)	0.0472 (8)	0.0358 (8)	−0.0119 (7)	−0.0016 (7)	−0.0172 (7)
S21	0.0551 (3)	0.0707 (3)	0.0517 (3)	0.0034 (2)	−0.0019 (2)	−0.0334 (2)
N23	0.0555 (10)	0.0538 (8)	0.0313 (7)	−0.0079 (7)	−0.0036 (6)	−0.0114 (6)
C32	0.0733 (14)	0.0818 (14)	0.0308 (8)	−0.0207 (11)	−0.0003 (9)	−0.0119 (8)

### Geometric parameters (Å, º)

**Table:**

C1—N1	1.2851 (19)	C12—H12E	0.9800
C1—C10	1.477 (2)	C12—H12F	0.9800
C1—C2	1.499 (2)	C21—N21	1.2863 (19)
C2—C3	1.541 (3)	C21—C30	1.475 (2)
C2—C3'	1.560 (9)	C21—C22	1.504 (2)
C2—H2A	0.9900	C22—C23	1.543 (3)
C2—H2B	0.9900	C22—C23'	1.584 (4)
C2—H2C	0.9900	C22—H22A	0.9900
C2—H2D	0.9900	C22—H22B	0.9900
C3—C4	1.519 (8)	C22—H22C	0.9900
C3—H3A	0.9900	C22—H22D	0.9900
C3—H3B	0.9900	C23—C24	1.412 (4)
C4—C5	1.540 (6)	C23—H23A	0.9900
C4—H4A	0.9900	C23—H23B	0.9900
C4—H4B	0.9900	C23'—C24	1.380 (4)
C3'—C4'	1.45 (3)	C23'—H23C	0.9900
C3'—H3C	0.9900	C23'—H23D	0.9900
C3'—H3D	0.9900	C24—C25	1.502 (3)
C4'—C5	1.41 (3)	C24—H24A	0.9900
C4'—H4C	0.9900	C24—H24B	0.9900
C4'—H4D	0.9900	C24—H24C	0.9900
C5—C6	1.395 (2)	C24—H24D	0.9900
C5—C10	1.399 (2)	C25—C26	1.390 (3)
C6—C7	1.370 (3)	C25—C30	1.401 (2)
C6—H6	0.9500	C26—C27	1.375 (3)
C7—C8	1.389 (3)	C26—H26	0.9500
C7—H7	0.9500	C27—C28	1.377 (3)
C8—C9	1.381 (2)	C27—H27	0.9500
C8—H8	0.9500	C28—C29	1.374 (3)
C9—C10	1.398 (2)	C28—H28	0.9500
C9—H9	0.9500	C29—C30	1.400 (2)
N1—N2	1.3727 (18)	C29—H29	0.9500
N2—C11	1.362 (2)	N21—N22	1.373 (2)
N2—H2	0.90 (2)	N22—C31	1.366 (2)
C11—N3	1.320 (2)	N22—H22	0.88 (2)
C11—S1	1.6841 (17)	C31—N23	1.324 (2)
N3—C12	1.448 (2)	C31—S21	1.6816 (18)
N3—H3	0.88 (2)	N23—C32	1.455 (2)
C12—H12A	0.9800	N23—H23	0.85 (2)
C12—H12B	0.9800	C32—H32A	0.9800
C12—H12C	0.9800	C32—H32B	0.9800
C12—H12D	0.9800	C32—H32C	0.9800
			
N1—C1—C10	116.87 (13)	H12D—C12—H12F	109.5
N1—C1—C2	123.86 (14)	H12E—C12—H12F	109.5
C10—C1—C2	119.27 (13)	N21—C21—C30	116.47 (13)
C1—C2—C3	113.13 (16)	N21—C21—C22	123.90 (14)
C1—C2—C3'	110.1 (4)	C30—C21—C22	119.63 (13)
C1—C2—H2A	109.0	C21—C22—C23	111.25 (18)
C3—C2—H2A	109.0	C21—C22—C23'	111.96 (18)
C1—C2—H2B	109.0	C21—C22—H22A	109.4
C3—C2—H2B	109.0	C23—C22—H22A	109.4
H2A—C2—H2B	107.8	C23'—C22—H22A	136.4
C1—C2—H2C	109.6	C21—C22—H22B	109.4
C3—C2—H2C	134.0	C23—C22—H22B	109.4
C3'—C2—H2C	109.6	C23'—C22—H22B	70.4
C1—C2—H2D	109.6	H22A—C22—H22B	108.0
C3'—C2—H2D	109.6	C21—C22—H22C	109.2
H2A—C2—H2D	136.7	C23—C22—H22C	70.8
H2C—C2—H2D	108.2	C23'—C22—H22C	109.2
C4—C3—C2	109.4 (4)	H22B—C22—H22C	137.9
C4—C3—H3A	109.8	C21—C22—H22D	109.2
C2—C3—H3A	109.8	C23'—C22—H22D	109.2
C4—C3—H3B	109.8	H22C—C22—H22D	107.9
C2—C3—H3B	109.8	C24—C23—C22	113.7 (2)
H3A—C3—H3B	108.2	C24—C23—H23A	108.8
C3—C4—C5	106.5 (4)	C22—C23—H23A	108.8
C3—C4—H4A	110.4	C24—C23—H23B	108.8
C5—C4—H4A	110.4	C22—C23—H23B	108.8
C3—C4—H4B	110.4	H23A—C23—H23B	107.7
C5—C4—H4B	110.4	C24—C23'—C22	113.0 (3)
H4A—C4—H4B	108.6	C24—C23'—H23C	109.0
C4'—C3'—C2	109.1 (11)	C22—C23'—H23C	109.0
C4'—C3'—H3C	109.9	C24—C23'—H23D	109.0
C2—C3'—H3C	109.9	C22—C23'—H23D	109.0
C4'—C3'—H3D	109.9	H23C—C23'—H23D	107.8
C2—C3'—H3D	109.9	C23'—C24—C25	117.8 (2)
H3C—C3'—H3D	108.3	C23—C24—C25	114.3 (2)
C5—C4'—C3'	116.9 (19)	C23'—C24—H24A	133.1
C5—C4'—H4C	108.1	C23—C24—H24A	108.7
C3'—C4'—H4C	108.1	C25—C24—H24A	108.7
C5—C4'—H4D	108.1	C23—C24—H24B	108.7
C3'—C4'—H4D	108.1	C25—C24—H24B	108.7
H4C—C4'—H4D	107.3	H24A—C24—H24B	107.6
C6—C5—C10	118.82 (16)	C23'—C24—H24C	107.9
C6—C5—C4'	121.1 (11)	C25—C24—H24C	107.9
C10—C5—C4'	118.1 (11)	H24B—C24—H24C	141.8
C6—C5—C4	120.9 (3)	C23'—C24—H24D	107.9
C10—C5—C4	120.2 (3)	C23—C24—H24D	137.6
C4'—C5—C4	16.3 (11)	C25—C24—H24D	107.9
C7—C6—C5	121.20 (17)	H24C—C24—H24D	107.2
C7—C6—H6	119.4	C26—C25—C30	119.05 (17)
C5—C6—H6	119.4	C26—C25—C24	121.30 (17)
C6—C7—C8	120.10 (16)	C30—C25—C24	119.65 (16)
C6—C7—H7	119.9	C27—C26—C25	121.11 (19)
C8—C7—H7	119.9	C27—C26—H26	119.4
C9—C8—C7	119.78 (16)	C25—C26—H26	119.4
C9—C8—H8	120.1	C26—C27—C28	120.03 (17)
C7—C8—H8	120.1	C26—C27—H27	120.0
C8—C9—C10	120.45 (16)	C28—C27—H27	120.0
C8—C9—H9	119.8	C29—C28—C27	120.02 (18)
C10—C9—H9	119.8	C29—C28—H28	120.0
C9—C10—C5	119.64 (14)	C27—C28—H28	120.0
C9—C10—C1	120.94 (14)	C28—C29—C30	120.82 (17)
C5—C10—C1	119.41 (14)	C28—C29—H29	119.6
C1—N1—N2	117.98 (13)	C30—C29—H29	119.6
C11—N2—N1	119.22 (14)	C29—C30—C25	118.97 (15)
C11—N2—H2	117.8 (13)	C29—C30—C21	120.96 (14)
N1—N2—H2	123.0 (13)	C25—C30—C21	120.07 (14)
N3—C11—N2	115.75 (14)	C21—N21—N22	117.99 (13)
N3—C11—S1	124.70 (12)	C31—N22—N21	118.72 (14)
N2—C11—S1	119.54 (13)	C31—N22—H22	115.9 (14)
C11—N3—C12	124.39 (15)	N21—N22—H22	123.3 (14)
C11—N3—H3	117.7 (14)	N23—C31—N22	115.30 (16)
C12—N3—H3	117.9 (14)	N23—C31—S21	124.95 (13)
N3—C12—H12A	109.5	N22—C31—S21	119.74 (13)
N3—C12—H12B	109.5	C31—N23—C32	123.87 (18)
H12A—C12—H12B	109.5	C31—N23—H23	120.0 (15)
N3—C12—H12C	109.5	C32—N23—H23	116.0 (15)
H12A—C12—H12C	109.5	N23—C32—H32A	109.5
H12B—C12—H12C	109.5	N23—C32—H32B	109.5
N3—C12—H12D	109.5	H32A—C32—H32B	109.5
N3—C12—H12E	109.5	N23—C32—H32C	109.5
H12D—C12—H12E	109.5	H32A—C32—H32C	109.5
N3—C12—H12F	109.5	H32B—C32—H32C	109.5

### Hydrogen-bond geometry (Å, º)

**Table:**

*D*—H···*A*	*D*—H	H···*A*	*D*···*A*	*D*—H···*A*
N3—H3···S21^i^	0.88 (2)	3.03 (2)	3.7226 (15)	138.0

Symmetry code: (i) −*x*+1, −*y*+1, −*z*+1.
